# Korean Triage and Acuity Scale education using role-playing and its effects on triage competency: A quasi-experimental design

**DOI:** 10.1371/journal.pone.0311892

**Published:** 2024-10-14

**Authors:** Yon Hee Seo, Sun-Og Lim

**Affiliations:** 1 Department of Nursing Science, Andong National University Andong-si, Andong-si, Gyeongsangbuk-do, South Korea; 2 Department of Nursing, Shinsung University, Dangjin-si, Chungcheongnam-do, South Korea; PLoS ONE, UNITED STATES OF AMERICA

## Abstract

Triage is the process of prioritizing patients in the emergency department (ED). This has a pivotal role in ensuring patient safety and that the ED operates smoothly; therefore, triage training education is an important aspect of triage nurses’ preparedness in different emergency situations. This study employed a quasi-experimental research design using a non-equivalent control group pre–post-test design to verify the effect of the Korean Triage and Acuity Scale (KTAS) education using role-playing on the knowledge of triage, triage performance ability, and triage competency in nursing students. Participants were 78 fourth-year nursing students from Shinseong University in South Korea; 39 were assigned to the experimental group, and 39 to the control group. The intervention was conducted for three weeks, from January 31 to February 16, 2024. The experimental group received KTAS education through role-playing, whereas the control group received triage education through lectures. Triage knowledge (t = 2.94, *p* = .004) and triage performance ability (t = 3.11, *p* = .003) were significantly improved in the experimental group. However, there was no significant difference in triage competency between groups (t = 1.32, *p* = .192). The post-test score of triage record in the experimental group showed a significant improvement over time (t = 0.56, *p* < .001). This study demonstrated that KTAS education using role-playing was more effective in improving triage knowledge and performance ability than traditional lecture methods for nursing students. Triage education programs should be developed considering the effectiveness of various teaching methods.

## Introduction

Globally, emergency departments (EDs) face the critical issue of overcrowding, which is largely attributed to an influx of non-emergency cases. Overcrowding in EDs strains resources, delays urgent treatment, and hampers service efficiency [1]. To ensure quick and accurate care for critical cases, a robust triage system and training for triage nurses based on the severity of the patient’s condition is vital.

The Korean Triage and Acuity Scale (KTAS), which is used in EDs, was developed based on the Canadian Triage and Acuity Scale and has been used in emergency medical centers in South Korea since 2016 [2]. Triage is a critical and challenging task performed by ED nurses [3, 4]; however, systematic training programs that can improve the triage decision-making skills of triage nurses in South Korea are not available.

Currently, KTAS training primarily comprises theoretical explanations of the field applications of the KTAS algorithm. Education (lecture method) is mainly verbal-based or lecturer-centered and takes four to five hours per session using four triage cases. With this approach, learners may not be engaged, and the instructor is mostly in charge of creating educational activities. The current approach is insufficient for enhancing the decision-making skills of triage nurses in emergency settings [5], and lacks a process for objectively evaluating triage competency within the KTAS framework [2, 6]. In addition, studies in several countries have shown that nurses often lack adequate education and preparation in triage areas, including Sweden [7], Iran [8], South Korea [9], and Australia [10]. The latter study revealed that 42% of nurses had not been trained for triage, and 14% were not adequately prepared, even after attending triage education classes [10]. Nurses are responsible for triage, and they must be equipped for and trained in this role [11, 12]. Triage nurses will not employ triage correctly if they do not receive adequate and efficient education [8]. Nursing faculties need to expose nursing students to critical situations to prepare them for triage [13].

Various methods have been used in triage education, such as lectures, reading manuals (guidelines), simulations, workshops, and seminars; some of these could have advantages over others [13]. In parts of nursing education where there may be a lack of practical experience, it is important to utilize experiential learning methods, such as simulation and role-playing [14–17]. Role-playing in nursing education is a well-known teaching method in which learners acquire new roles or behaviors through training in a situation similar to real life by simulating actual patient situations for severity classification [18]. Introducing role-playing into KTAS education will significantly help nursing students understand and accurately identify real-life medical situations and patient problems. This method enhances their theoretical knowledge of triage and improves their decision-making and emergency treatment abilities, particularly for those lacking clinical experience.

We thus developed KTAS education using role-playing and tested its impact on the triage competency of nursing students before applying it to actual triage nurses in clinical settings. The objective of this study was to determine and compare the effectiveness of two methods, role-playing and lectures, on the triage knowledge, triage performance and triage competency of nursing’s students.

## Materials and methods

### Research design

This study used a non-equivalent control group pre–post-test design to verify the effect of KTAS education using role-playing on nursing students’ knowledge of triage, triage performance ability, and triage competency.

### Participants

Participants were recruited from the nursing departments of the universities of Shinseong, located in Dangjin-si, South Korea, after posting a recruitment notice on the nursing notice board and community explaining the study purpose and contents. All participants who agreed to participate were selected from among those who understood the study purpose, provided written consent, and met the study selection criteria: (1) completed the fundamentals of nursing and health assessment within the Department of Nursing Science, (2) had no previous experience with triage education, and (3) participated in adult nursing clinical practice as a fourth-year nursing student.

The experimental group was assigned to receive KTAS education using role-playing, whereas the control group was assigned to students who participated in triage education based on lectures (Table 1). Participants were not provided with information regarding the group to which they belonged. The group assignment method involved using number cards labelled with two groups. Participants who drew ’0’ were assigned to the experimental group, and those who drew ’1’ were assigned to the control group.

**Table 1:** Program contents based on role-playing learning.

Session (time)		Contents
1	Orientation (60 mins)	KTAS learning methods and procedures
		Role-playing learning method and procedure guide
		Guide to composition and role of team members
2	The concept of KTAS (120 mins)	Importance of KTAS in Emergency Situations
		Understanding the Need for KTAS
		How to perform KTAS
		Measuring the severity/urgency score according to the KTAS algorithm
3	Role-playing (120 mins)	Understanding eight emergency cases and your role based on the case
		Four cases allocated to each team randomly for role-playing
		Peer feedback and debriefing

KTAS = Korean Triage and Acuity Scale

The sample size was calculated with G*Power 3.1.9.4. According to a meta-analysis of virtual patient education n for health professionals, the effect size d was 0.8 [19]. In a two-tailed significance test with a power of 80% and an alpha level of .05, the sample size of each group was calculated to be 26. We recruited 78 participants (39 in the experimental group and 39 in the control group), considering a dropout rate.

### Ethical considerations

This study was conducted after Institutional Review Board approval (1040191-202401-HR-002-01). Written informed consent was obtained from all participants. All methods were conducted in accordance with the relevant guidelines and regulations.

### KTAS

**Fig 1:**
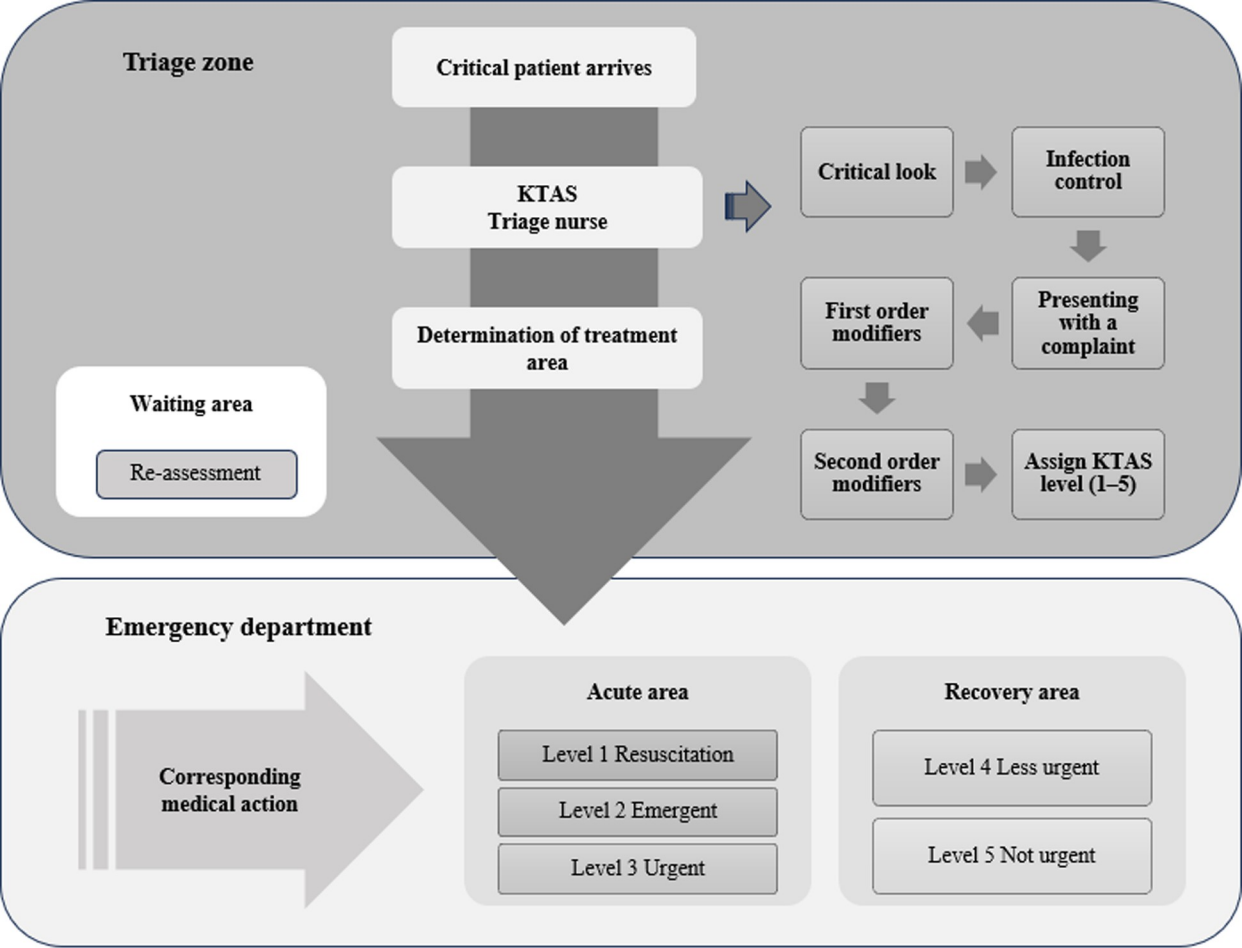
KTAS process. Note. KTAS = Korea Triage Acuity Scale.

The KTAS is used as a nationwide triage tool to determine patients’ priority for treatment in EDs in South Korea. KTAS levels are categorized according to emergency symptoms, which are divided into grades 1–5 depending on severity (Fig 1). When a patient arrives at the ED, a critical first look and a screening test for infectious diseases are performed, following which primary modifiers or special modifiers are selected to assign an appropriate score. The KTAS includes 155 main complaints of adults and 165 complaints of children. After the complaints are selected, the common and general characteristics of all complaints are the primary considerations, such as respiration status, vital signs, mental status, and pain. Secondary considerations include the specific characteristics of each complaint. These include blood sugar levels measured at the time of visit, symptoms of dehydration, high blood pressure, pregnancy, and mental health as judged by medical history and patients’ responses. The KTAS includes both severity and urgency [20]. Priority of care is determined based on the KTAS classification results.

### Measures

#### Demographic characteristics

Participants’ demographic characteristics included sex, age, grade point average (GPA), and major satisfaction. The scores on the major satisfaction tools ranged from 3 (“satisfied”) to 1 (“dissatisfied”). The higher the score, the higher the satisfaction with their major.

#### Triage knowledge measurement

The triage knowledge measurement tool was developed with reference to the Education Guidelines of Emergency Patients’ Classification and KTAS committees [2]. The expert group in this study comprised two ED triage nurses and three nursing professors who evaluated the validity of the triage knowledge questionnaire using content validity indices (CVI). The overall CVI on the triage knowledge questionnaire was 0.9. This tool comprises 20 items, including 3 items on the role of triage nurses and triage records, 11 items on the triage classification process and triage level, and 6 items on the primary consideration in performing KTAS. For each item, a score of 1 point was given for a correct answer and 0 points for an incorrect answer, resulting in a score range of 0–20 points. Higher scores indicated higher triage knowledge.

#### Triage performance ability measurement

Triage performance ability means that a triage/urgent triage role collects subjective and objective information and history about the patient to determine the severity/urgency of their condition, and then measures the severity/urgency score according to the KTAS algorithm. The triage performance ability measurement tool was developed with reference to the Education Guidelines of Emergency Patient Classification and KTAS Committees [2]. The researchers developed cases for four diseases—cardiovascular, respiratory, neurological, and gastrointestinal diseases—to assess triage performance ability. Each case component included age, gender, reason for visit, symptoms (including level of consciousness), and signs (vital signs and oxygen saturation). This tool comprises 10 items, with three items for KTAS Level 1, three items for KTAS Level 2, two items for KTAS Level 3, and two items for KTAS Level 4. The expert group in this study comprised two triage nurses in the ED and three nursing professors who evaluated the validity of the triage performance ability questionnaire using the CVI. The overall CVI score on the questionnaire was 0.90. The evaluation method required participants to read the case and write down the triage stage. For each item, a score of 1 point was given for a correct answer and 0 points for an incorrect answer, resulting in a score range of 0–10 points. A higher score indicated greater triage performance ability.

#### Triage competency measurement

Triage competency is the ability to allocate medical resources efficiently by determining care priorities according to patients’ health status [21]. This study used the triage competency measurement tool developed by Moon and Park [5]. This tool comprises 30 items divided into five sub-areas: clinical judgment (13 items), expert assessment (4 items), management of medical resources (4 items), timely decisions (4 items), and communication (5 items). Each item was measured on a Likert scale ranging from 0 (“not at all”) to 4 (“always”), with the total possible points ranging from 0 to 120. Higher scores indicate greater triage competency. The reliability at the time of development of this tool was Cronbach’s α .91 [5], and Cronbach’s α was .95 in this study, indicating excellent internal consistency.

#### Triage record measurement

The triage record measurement tool was developed with reference to the educational guidelines of the emergency patient classification and KTAS committees [2]. The expert group in this study comprised two triage nurses in the ED and three nursing professors who evaluated the validity of the triage record sheets using the CVI. The overall CVI score on the triage sheet was 0.91. This tool comprises 12 items, including 3 items on patient information; 7 items on patient assessment; and 2 items on the accuracy of the KTAS level, including the rationale for the triage level decision. For each item, a score of 2 points was given for an accurate record, 1 point for an average record, and 0 points for a poor or no record (score range = 0–24 points). A higher score indicated greater accuracy of the triage record.

### Data collection and experimental treatment

The intervention was conducted for three weeks, from January 31 to February 16, 2024. The experimental group received KTAS education through role-playing, whereas the control group received triage education through lectures. As shown in Table 1, the experimental and control groups received the same learning content and instructor guidance time for each session.

In the first week, an orientation was conducted, and training methods for role-playing were explained in terms of the KTAS learning process and procedures. In the second week, theoretical learning for KTAS was conducted. The training lasted 120 min and covered learning objectives, basics of severity classification, application of KTAS in adults, and the levels and methods of using KTAS. In the third week, training was conducted using emergency patient cases developed with reference to the education guidelines of the emergency patient classification and KTAS committees. Eight cases were chosen from the 17 disease categories, focusing on 4 categories with a high number of severe cases: cardiovascular, respiratory, digestive, and neurological. The cases included essential information for emergency classification, such as age, gender, reason for visit, symptoms (including consciousness level), and signs (vital signs and oxygen saturation), and they were structured to allow the triage level to be selected from 1 to 5.

For the experimental group that received KTAS education using role-playing, the teams comprised four members each. Each team member took turns playing the roles of patient, triage nurse, ER administrator, and triage facilitator so that everyone could experience all roles. The patient’s role was to become familiar with the relevant clinical case and express the motivation for admission and symptoms in a realistic manner, as if the patient had actually visited the ED. The role of the triage nurse was to directly perform a critical first look and select the primary modifiers examined when a patient arrives at the ED, then decide the triage level based on the results of the assessment and write the details of the triage level classification on the triage record. The role of the ED administrator was to help the triage nurses input the triage progress stages into the computer. The triage facilitator checked the triage monitoring record sheet to ensure that the triage nurse classified the patient using the correct method and KTAS algorithm and played a role in coordinating the overall work within the ED. When the role-playing learning for one case was completed, the triage record sheet submitted by each team was checked, and peer feedback was provided to determine whether the triage level was accurately classified. Every participant in this discussion was given the opportunity to share their thoughts, feelings, observations, and recommendations. Finally, each participant was asked to write a reflection journal about their role-playing and then move on to the next clinical case.

The control group received KTAS education using traditional lecture-style. In the first week, a 60-minute orientation covered training methods and program procedures. The second week featured a 120-minute lecture on KTAS concepts and algorithms for the control group. Subsequently, in the third week, the control group received case-based KTAS education via traditional lectures, and were trained to independently assess patient cases and determine triage levels. The control group received eight cases from four categories with a high number of severe cases: cardiovascular, respiratory, digestive, and neurological, which were the same as the experimental group. Following individual KTAS learning using the eight cases, the instructor provided feedback to each student regarding the accuracy of their triage level determination.

To minimize participant contamination, the control group received KTAS education in the morning in a conventional classroom setting for theory lectures. In contrast, the experimental group received KTAS training in the afternoon in a problem-based learning environment conducive to team-based learning. To minimize the diffusion of the educational intervention, the classrooms of the control and experimental groups were housed in different buildings. The program schedule ensured that the control group received KTAS training via traditional lectures first, followed by role-playing sessions for the experimental group.

### Data analysis

Collected data were analyzed using SPSS 25.0 (IBM, Chicago, IL, USA). Participants’ general characteristics were analyzed as numbers, percentages, means, and standard deviations. To verify the homogeneity of the experimental group and the control group, χ^2^-tests and t-tests were performed. To verify the differences in triage knowledge, performance, and competency, we calculated the difference between the pre-post means within each group and then compared the differences between the two groups using an independent t-test. As the pre-test result presented a difference in baseline triage knowledge, an analysis of covariance was applied to verify the difference in post-knowledge between the two groups using pre-knowledge as a covariate.

## Results

### Homogeneity test for general characteristics and dependent variables

The general characteristics of the experimental and control groups and homogeneity tests are presented in Table 2. There were no significant differences in gender, GPA, or satisfaction with nursing major between groups (*p* > .05). Among the dependent variables, triage knowledge, performance ability, and competency showed homogeneity between groups (*p* > .05).

**Table 2:** Homogeneity test for general characteristics and the dependent variables (N = 78).

Variable	Category	EG (n = 39)	CG (n = 39)	χ^2^ or t	*p*
		n (%) or Mean ± SD	n (%) or Mean ± SD		
Age (years)		25.82 ± 7.91	25.44 ± 6.89		
Gender	Woman	37 (47.4)	33 (42.3)	2.23	.135
	Man	2 (2.6)	6 (7.7)		
Grade point average	≥ 4.0	8 (10.3)	4 (5.1)	1.63	.443
	2.0–3.9	28 (35.9)	31 (39.7)		
	< 2.0	3 (3.8)	4 (5.1)		
Nursing major satisfaction	Extremely Satisfied	33 (42.3)	33 (42.3)	0.44	.801
	Moderately Satisfied	5 (6.4)	4 (5.1)		
	Dissatisfied	1 (1.3)	2 (2.6)		
Triage knowledge		11.36 ± 1.84	11.46 ± 2.12	0.23	.167
Triage performance ability		2.92 ± 1.56	3.15 ± 1.23	0.77	.456
Triage competency		2.88 ± 0.46	2.98 ± 0.40	0.92	.233

EG = experimental group; CG = control group; SD = standard deviation

### Pre- and post-comparison of triage knowledge and triage performance ability between the groups

The first hypothesis in this study posited that the experimental group would show higher triage knowledge than those in the control group. The experimental group’s pre-post triage knowledge score showed significant improvement compared with that of the control group (t = 2.944, *p* = .004).

The second hypothesis was that the experimental group would show higher triage performance ability than the control group. When the pre-post triage performance ability of the two groups was verified, the experimental group showed significant improvement (t = 3.106, *p* = .003). Specifically, the results of analyzing the performance ability in KTAS level showed that there was a significant difference in the KTAS Level 2 “emergency” between groups (t = 2.363, *p* = .021; Table 3).

**Table 3:** Comparison of triage knowledge and triage performance ability between the groups.

Variable	EG (n = 39)		CG (n = 39)		t	*p*
	Pre-test	Post-test	Pre-test	Post-test		
	Mean ± SD		Mean ± SD			
Triage knowledge	11.36 ± 0.32	15.41 ± 0.35	11.46 ± 0.32	13.95 ± 0.35	2.944	.004
Triage performance ability	2.92 ± 0.23	5.31 ± 0.24	3.15 ± 0.23	4.26 ± 0.24	3.106	.003
Resuscitation L1	0.59 ± 0.11	0.56 ± 0.11	0.56 ± 0.11	0.49 ± 0.11	0.485	.629
Emergency L2	0.82 ± 0.12	1.69 ± 0.14	0.80 ± 0.12	0.97 ± 0.14	2.363	.021
Urgency L3	0.92 ± 0.14	1.67 ± 0.16	1.00 ± 0.14	1.31 ± 0.16	1.210	.230
Less or no urgency L4–5	0.59 ± 0.11	1.39 ± 0.10	0.80 ± 0.11	1.31 ± 0.10	0.527	.600

EG = experimental group; CG = control group; SD = standard deviation; L = level

### Pre- and post-comparison of triage competency between the groups

The hypothesis in this study posited that the experimental group would show higher triage competency than the control group. There was no significant difference between the two groups regarding triage competency when the pre- and post-test values were compared (t = 1.316, *p* = .192). Specifically, after examining the sub-factors of the pre-post triage competency, the experimental group demonstrated significant improvement in expert assessment (t = 2.163, *p* = .034) and communication (t = 2.056, *p* = .043) compared to the control group (Table 4).

**Table 4:** Comparison of triage competency and triage record score between the groups.

Variable	EG (n = 39)		CG (n = 39)		t	*p*
	Pre-test	Post-test	Pre-test	Post-test		
	Mean ± SD		Mean ± SD			
Triage competency	2.89 ± 0.07	3.49 ± 0.06	2.98 ± 0.07	3.37 ± 0.06	1.316	.192
Clinical judgment	2.86 ± 0.08	3.46 ± 0.07	3.01 ± 0.08	3.41 ± 0.07	0.510	.611
Expert assessment	2.83 ± 0.09	3.60 ± 0.07	2.86 ± 0.09	3.38 ± 0.07	2.163	.034
Management of medical resources	2.76 ± 0.10	3.42 ± 0.09	2.81 ± 0.10	3.33 ± 0.09	0.679	.499
Timely decisions	2.69 ± 0.10	3.30 ± 0.83	2.66 ± 0.10	3.21 ± 0.83	0.764	.447
Communication	3.24 ± 0.87	3.71 ± 0.84	3.35 ± 0.87	3.46 ± 0.84	2.056	.043

EG = experimental group; CG = control group; SD = standard deviation

### Comparison of triage record score in the only experimental group over time

The comparison of the triage record scores of the experimental group over time showed a significant difference, with the average score increasing from 17.08 ± 2.45 at the first recording to 19.26 ± 2.06 at the second recording (t = 0.561, *p* < .01). Post examining the sub-factors of the triage record score over time, significant differences were observed in the patient assessment (t = 4.257, *p* < .01), main symptom (t = 2,512, *p* = .014), vital signs/SpO^2^ (t = 6.183, *p* < .01), pain assessment (t = 3.411, *p* = .002), accident mechanism (t = 2.695, *p* = .010), and the accuracy of triage records (t = 5.707, *p* < .001; Table 5).

**Table 5:** Comparison of triage record score in the experimental group over times (n = 39).

Variable	1st triage recode	2nd triage recode	Mean difference	t (*p*)
	Mean ± SD	Mean ± SD	Mean ± SD	
Triage record score	17.08 ± 2.45	19.26 ± 2.06	2.17 ± 2.16	0.561 (.001)
Patient information	1.29 ± 0.39	1.42 ± 0.21	0.13 ± 0.33	2.427 (.20)
Patient assessment	1.45 ± 0.25	1.62 ± 0.21	0.17 ± 0.25	4.257 (.001)
Main symptom	1.74 ± 0.64	2.00 ± 0.00	0.25 ± 0.64	2,512 (.014)
Subjective evaluation	1.62 ± 0.63	1.79 ± 0.62	0.18 ± 0.72	1.555 (.128)
GCS assessment	1.85 ± 0.43	1.92 ± 0.35	0.08 ± 0.58	0.829 (.412)
Vital sign/SpO^2^	0.97 ± 0.54	1.72 ± 0.46	0.74 ± 0.75	6.183 (.001)
Pain assessment	1.74 ± 0.60	1.33 ± 0.53	0.41 ± 0.75	3.411 (.002)
Underlying disease	1.21 ± 0.52	1.21 ± 0.41	0.00 ± 0.65	0.000 (1.000)
Accident mechanism	1.03 ± 0.50	1.36 ± 0.71	0.33 ± 0.77	2.695 (.010)
Accuracy of triage records	1.53 ± 0.38	1.83 ± 0.35	0.31 ± 0.34	5.707 (.001)

GCS = Glasgow Coma Scale; SD = standard deviation

## Discussion

This study was conducted to verify the effects of KTAS education using role-playing on knowledge of triage, triage performance ability, and triage competency, and to provide evidence for establishing a foundation for emergency patient classification education in future nursing education.

The experimental group that received KTAS education using role-playing showed significantly higher triage knowledge than the lecture group. Role-play may be more effective for triage training than lecture methods [14, 18, 22, 23], which coincides with our results. Considering that students have not been using role-playing teaching methods thus far, Heidarzadeh et al. [23] observed nursing students’ learning motivation and the collaborative environment in a role-play group, which might contribute to their willingness to learn triage knowledge. Conversely, traditional lecture methods place learners in a passive role, limiting their opportunities for collaboration [22]. This study showed that triage knowledge improved when KTAS education using role-playing was applied to nursing students.

The experimental group showed a significantly higher triage performance ability than the control group. This result coincides with those of previous studies on triage among nursing students [13, 23]. In addition, analysis of the triage performance ability sub-area revealed a significant difference in KTAS Level 2 between the groups in this study. If the KTAS education using role-playing can help the nursing students in identifying life-threatening cases better like “severe emergency patients” (KTAS Level 2), they will be more beneficial. Role play is recognized as a good way to boost performance because it provides a stress-free, safe atmosphere and repeated assessment practice for gaining expertise in clinical situations [18, 24]. Nursing students performed a 20-minute role-play several times based on each role that incorporated the concepts of the KTAS algorithm. In particular, the first impression of an emergency patient and a vivid expression of chief complaints, like a realistic patient, seem to make it easy to follow the process of the KTAS algorithm step-by-step. Nursing students also had the opportunity to share their opinions or be rationally related to KTAS-level decision-making and the error of KTAS algorithm application in the reflection phase. Through such feedback and debriefing, the difficulties in matching patients’ main complaints and the triage algorithm [4] have been improved. From this, it can be inferred that the triage performance ability of nursing students was enhanced.

This study confirmed the effect of applying KTAS education using role-playing on the triage competency of nursing students, and there was no difference from the control group that received lecture education. However, sub-factors such as “communication” and “expert assessment” significantly improved. Direct comparison is impossible because there are no similar studies on nursing students; however, our results contradict those of a previous study that measured the effect of nurses’ triage competency based on a competency-based triage education application [25]. Important factors to consider in relation to triage nurses’ triage competency are not only knowledge [4] and triage training [26], but also critical thinking disposition, problem-solving ability, and clinical judgment ability [5, 27]. However, the current KTAS education comprises a four-hour training program that includes lectures and cases covering the concept of KTAS, the application of KTAS in adult and pediatric patients, and the selection of relevant items from the first and second considerations to determine KTAS level. This section focuses on applying the KTAS algorithm to the presented cases. The content of education to improve critical thinking disposition, problem-solving ability, and clinical judgment ability, which are key factors in improving trial competency, was not considered. Given that these factors cannot be improved by short-term theoretical education, it appears that it was somewhat difficult to improve triage competency after KTAS training was conducted for nursing students without clinical experience by applying role-playing for a short period of three weeks. Therefore, to enhance the triage competency of nursing students, it is necessary to improve the current one-time education focusing on triage knowledge and the KTAS algorithm, and implement KTAS education from an undergraduate course that can comprehensively enhance the key elements suggested in a previous study [5, 26, 27]. According to Delnavaz et al. [13], combining the interactive educational methods can improve the learning of nursing students.

As expert assessments identify patients’ problems through physical examinations and interviews, physical assessment and communication require direct interaction between patients and nurses. Therefore, KTAS education using role-playing was more effective in those sub-areas (physical assessment and communication) compared to mobile-based non-face-to-face study [25] or the control group with lecture methods. Therefore, to improve nursing students’ triage competency, it is necessary to develop a program that combines various educational methods such as simulation, role-play, workshops, and seminars. These methods improve not only screening knowledge but also communication, physical assessment, and clinical reasoning.

In view of the rising importance of nursing records in legal disputes [28] and because nurse triage outcomes in South Korea are closely related to the cost of emergency medical care [29], evidence for triage decisions should be carefully documented. However, studies on nursing records in triage education are limited. Therefore, we analyzed the effect of triage records on nursing students receiving KTAS education through role-playing over time. KTAS education using role-playing improved the ability to write nursing records over time. Unlike traditional lecture-style KTAS education, the KTAS education method allows students to experience not only the understanding of triage algorithms but also the roles of ED colleagues, such as the ED administrator and the triage facilitator, through role-play. This may be helpful in writing accurate nursing records, even in tense situations in the ED, owing to feedback from colleagues. This greatly contributes to nursing students learning more accurate and effective ways to write ED nursing records through KTAS education. Future research should compare the effects of the two educational methods on the improvement of nursing records between the control and experimental groups.

This study has a few limitations. First, it should be noted that the majority of the nursing students (37 out of 39) who participated were female, indicating a gender distribution limitation. We measured the effectiveness of KTAS education through role-playing immediately after its use. Therefore, it was impossible to estimate the continuation of the educational effect on nursing students or the interval requiring repeated education. Further research is required to determine the sustainability of the effects of this program. This study was conducted in only one nursing school in South Korea; therefore, the generalizability of the results is limited.

## Conclusion

KTAS education using role-playing was more effective than lecture-style education in improving triage knowledge and performance ability. Although this was insufficient to improve the triage competency of nursing students, it can be concluded that education utilizing role-play is more effective in improving communication and expert assessment capabilities. In addition, this study is meaningful in that it utilized basic data on emergency nursing among nursing students, as it is the first study to conduct KTAS professional education for domestic nursing students.

## Supporting information

S1 Data set (XLSX)
